# Case Report: An ovarian epithelial-type serous adenocarcinoma of the testis was treated using systemic chemotherapy with bevacizumab, the treatment strategy used for ovarian serous carcinoma

**DOI:** 10.3389/fonc.2025.1467579

**Published:** 2025-03-21

**Authors:** Rui-Zhi Liu, Shuang Zhang, Jing Gong, Jin Yao, Hao Zeng, Lang He

**Affiliations:** ^1^ School of Medical and Life Sciences, Chengdu University of Traditional Chinese Medicine, Chengdu, China; ^2^ Department of Biotherapy, West China Hospital, West China Medical School, Sichuan University, Chengdu, Sichuan, China; ^3^ Department of Pathology, West China Hospital, West China Medical School, Sichuan University, Chengdu, Sichuan, China; ^4^ Department of Radiology, West China Hospital, West China Medical School, Sichuan University, Chengdu, Sichuan, China; ^5^ Department of Urology, West China Hospital, West China Medical School, Sichuan University, Chengdu, Sichuan, China; ^6^ Department of Oncology, Cancer Prevention and Treatment Institute of Chengdu, Chengdu Fifth People’s Hospital, Chengdu, Sichuan, China

**Keywords:** ovarian type tumors of testis, rare tumor, chemotherapy, bevacizumab, case report

## Abstract

Ovarian-type serous adenocarcinoma of the testis is an extremely rare malignant disease, and experience and evidence regarding its treatment are lacking. Herein, we describe the case of a 39-year-old man who presented with widespread metastasis in the retroperitoneal and pelvic lymph nodes after surgical resection of his right testicular tumor. Systemic chemotherapy with bevacizumab, the treatment strategy used for ovarian serous adenocarcinoma, was administered, and retroperitoneal lymph node dissection and radiotherapy followed after the lesions regressed. The patient has had over 3 years of progression-free survival. This is the first case of a bevacizumab combination in this disease and indicates that bevacizumab may be an option for the treatment strategy of this rare disease.

## Introduction

Ovarian-type tumors of the testis are extremely rare and resemble ovarian surface epithelial tumors (1). Although uncommon, their pathological types include serous, mucinous, endometrioid, and Brenner tumors, with the most frequently reported form being the serous subtype (2–4). The diagnosis of this disease is challenging due to limited clinical experience, and the treatment approach lacks solid evidence. Given its similarity to ovarian cancer, it is reasonable to apply the same treatment protocols used for ovarian carcinoma in women (1, 5, 6). However, few cases have been reported that detail the treatment and its effectiveness for these tumors. Here, we report a case of serous adenocarcinoma of the testis with widespread metastasis to the retroperitoneal and pelvic lymph nodes. After treatment with chemotherapy and bevacizumab, following the treatment strategy used for ovarian counterparts, the patient achieved long-term survival.

## Case report

**Figure 1:**
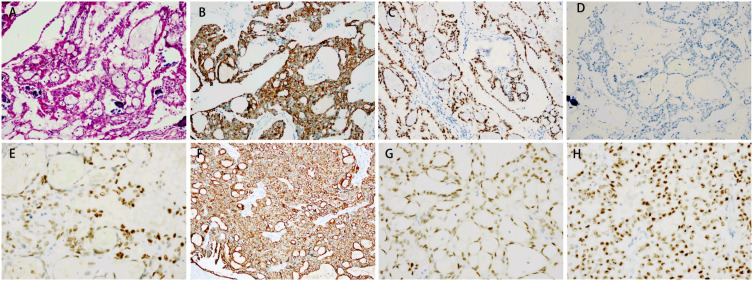
Immunohistochemical staining of the testis and lymph nodes. **(A)** Hematoxylin and eosin (HE) staining of the testis. Magnification: ×20. The testis tissue showed positive staining for CK7 **(B)** and PAX8 **(C)**, and negative staining for calretinin **(D)**. Magnification: ×40. The lymph node tissue showed positive staining for ER **(E)**, CK7 **(F)**, PAX8 **(G)** and WT-1 **(H)**.

**Figure 2:**
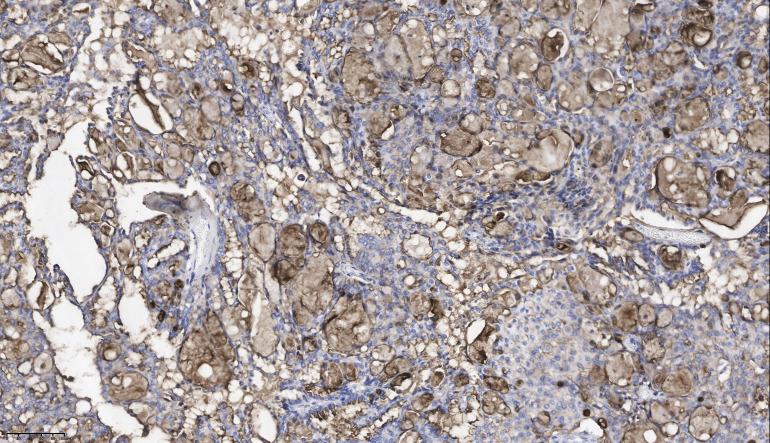
The lymph node tissue showed positive staining for CA-125. Magnification: ×20.

A 39-year-old man presented to our hospital in March 2021 with a complaint of a right testicular mass. The right testicular nodule had been present and stable for 6 years, but it had gradually grown to 10 cm at the time of consultation, with a mass also developing in his right groin. MRI revealed a right testicular mass with enlarged inguinal lymph nodes. Following radical resection of the right testicular tumor with right inguinal lymph node dissection (March 2021), the pathological diagnosis of the testicular mass was established as “ovarian-type tumor, serous adenocarcinoma,” with cancer also identified in four out of seven inguinal lymph nodes. This diagnosis was confirmed by immunohistochemical analysis from both our hospital and West China Hospital, showing positive staining for pan-CK, ER, CK7, Pax8, EMA, WT-1, and CA-125, and negative stains for D2-40, PLAP, SALL4, OCT4, Inhibin-a, Calretinin, CK20, and P63 (**Figures 1**, **2**, **Table 1**). However, more than one month later, a computed tomography (CT) scan at West China Hospital revealed widespread metastases in the retroperitoneal and pelvic lymph nodes, with an elevated serum CA-125 level (1,429 U/ml). After consulting with the patient and referring to the treatment strategy for ovarian serous adenocarcinoma, a chemotherapy regimen of paclitaxel and carboplatin combined with bevacizumab was administered for eight cycles from April to November 2021.

**Table 1:** Immunohistochemical and serum marker profiles in ovarian epithelial type serous adenocarcinoma of the testis.

Marker type	Marker	Result	Meaning
Immunohistochemistry (Positive)	pan-CK	Positive	Supports tumor of epithelial origin
	ER	Positive	Suggests the tumor may be sensitive to hormone therapy
	CK7	Positive	Commonly found in tumors of Müllerian duct origin
	PAX8	Positive	Marker for Müllerian duct differentiation; supports ovarian-type tumor diagnosis
	EMA	Positive	Epithelial membrane antigen positive; further supports tumor of epithelial origin
	WT-1	Positive	Wilms tumor protein 1 positive; commonly seen in ovarian serous carcinoma
Immunohistochemistry (Negative)	D2-40	Negative	Excludes tumors of lymphatic origin
	PLAP	Negative	Excludes germ cell tumor
	SALL4	Negative	Excludes germ cell tumor
	OCT4	Negative	Excludes germ cell tumor
	Inhibin-a	Negative	Excludes sex cord stromal tumor
	Calretinin	Negative	Excludes mesothelioma
	CK20	Negative	Excludes tumors of gastrointestinal origin
	P63	Negative	Excludes squamous cell differentiation tumor
Serum markers	CA-125	Elevated	Diagnostic and prognostic marker for ovarian-type serous carcinoma
	HE4	Elevated	May be used with CA-125 to improve diagnostic accuracy
	CEA	Elevated	May be elevated in certain epithelial tumors
	CA19-9	Elevated	May have a reference value in certain ovarian-type tumors
	AFP	Negative	Excludes yolk sac tumor or hepatocyte-differentiated tumor
	hCG	Negative	Excludes choriocarcinoma component

**Figure 3:**
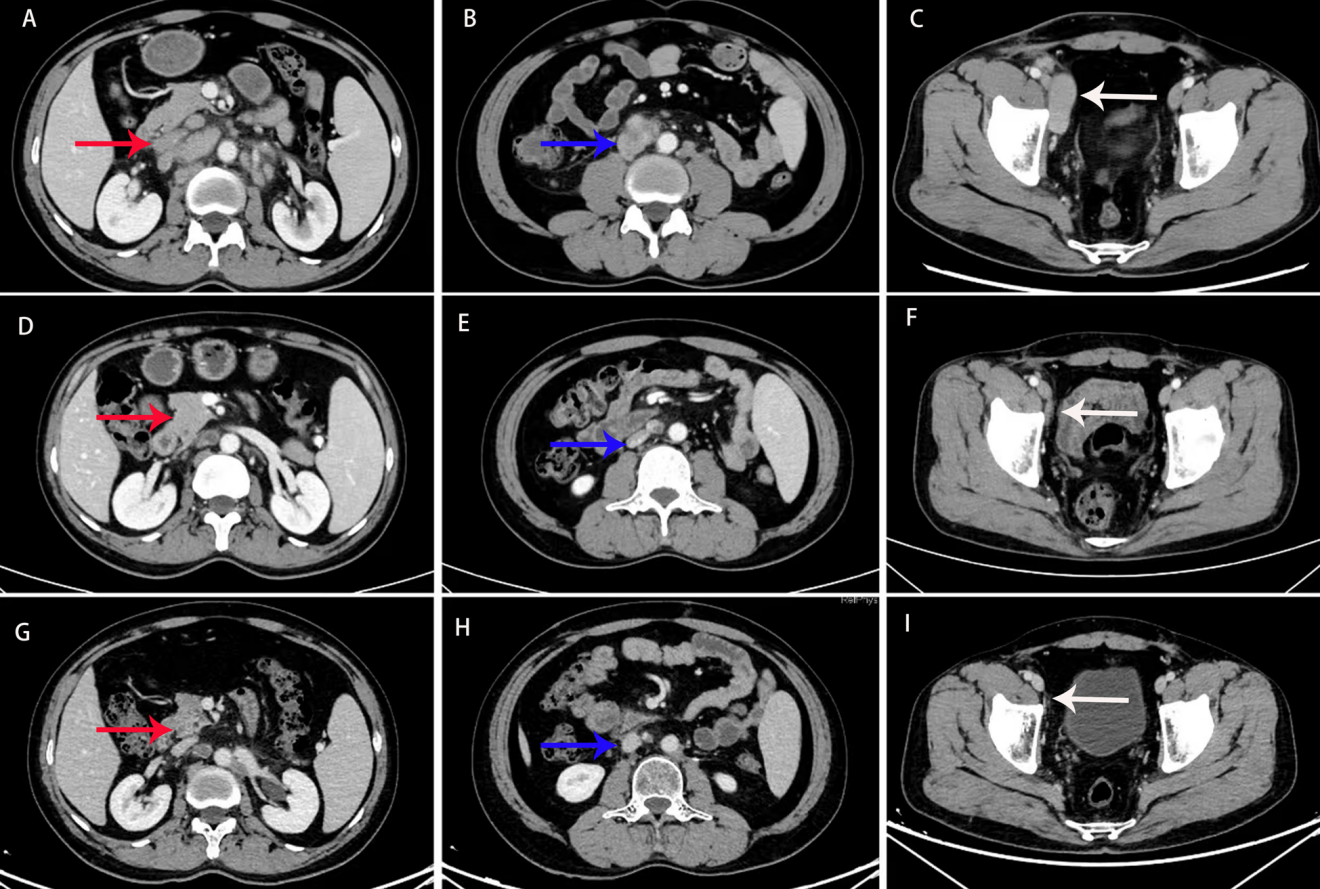
Computed tomography (CT) scan before treatment **(A–C),** and 6 **(D–F)** and 30 months **(G–I)** after treatment. CT imaging revealed extensive metastasis in the retroperitoneal and pelvic lymph nodes (indicated by the red, blue, and white arrows). After 6 and 30 months of treatment, the lesions showed a significant reduction, and the therapeutic response was evaluated as a partial response (PR).

**Figure 4:**
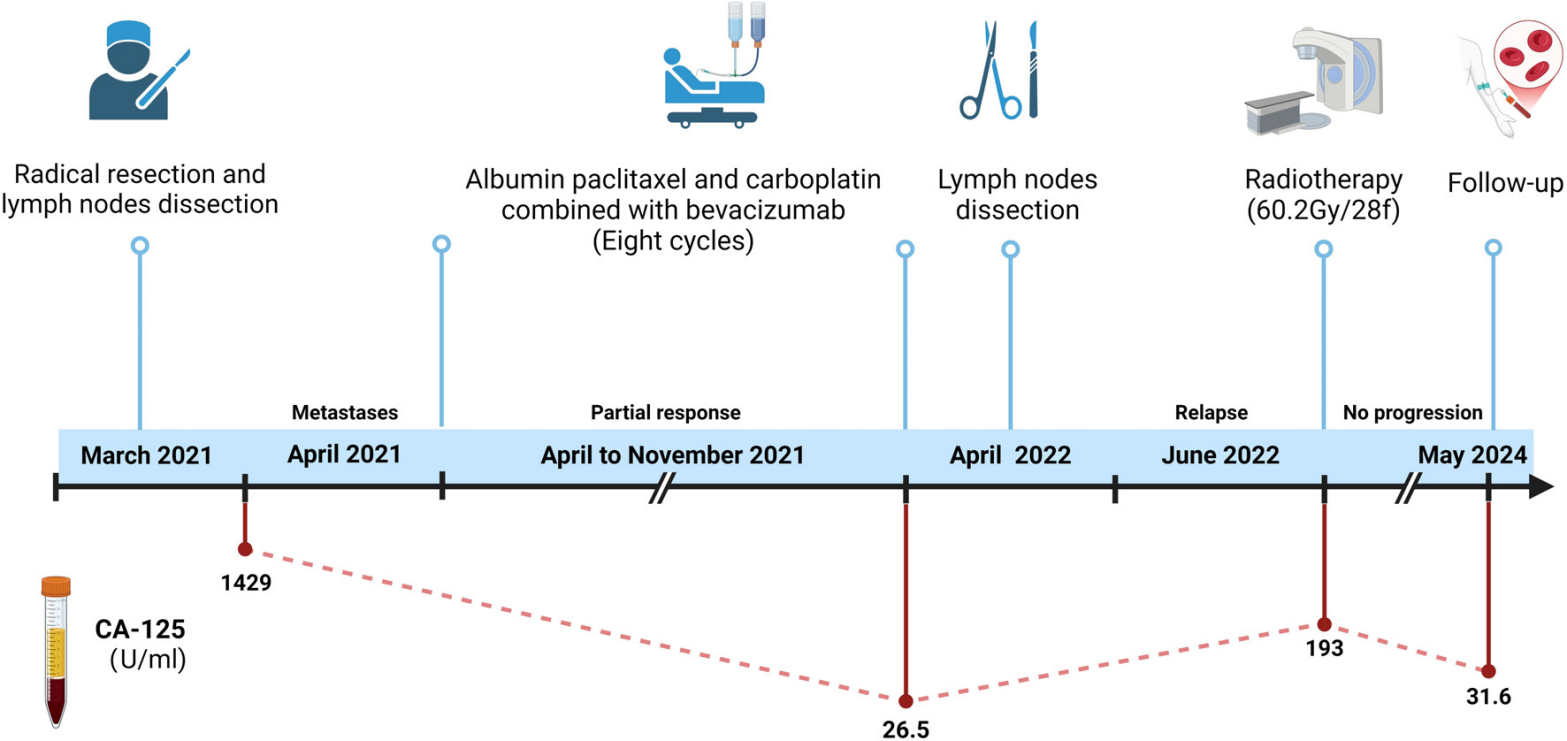
Treatment timeline. This figure illustrates the chronological sequence of the patient’s treatment. It includes the key events from the initial diagnosis in March 2021, the radical resection with lymph node dissection, the chemotherapy regimen (paclitaxel, carboplatin, and bevacizumab), the subsequent radiotherapy in June 2022, and the follow-up evaluations through May 2024. The dynamic change of CA-125 levels is also shown. (Created in https://BioRender.com).

The patient initially experienced leukopenia and gingivitis during paclitaxel chemotherapy. The leukopenia was managed with granulocyte colony-stimulating factor (G-CSF), resulting in an improvement, while the gingivitis was treated with acetylspiramycin, ornidazole, and chlorhexidine mouthwash, leading to a resolution of symptoms. After the second cycle of paclitaxel infusion, the patient developed flushing, palpitations, and shortness of breath, which were considered allergic reactions to paclitaxel. Consequently, the chemotherapy regimen was switched to albumin-bound paclitaxel. The patient tolerated albumin-bound paclitaxel and bevacizumab well, with no significant adverse reactions, including severe nausea, vomiting, leukopenia, hypertension, or bleeding. A partial response (PR) was achieved, with a reduction in serum CA-125 levels to 26.5 U/mL after chemotherapy combined with targeted therapy (**Figure 3**). Residual lesions with reduced metabolism were noted on PET/CT scan, and retroperitoneal lymph node dissection was performed in April 2022, with cancerous metastasis found in four out of nine lymph nodes, similar in pathological features to the testicular tumor. Although maintenance treatment was not adopted, the patient soon experienced a recurrence in the left supraclavicular lymph node, and radiotherapy was performed in June 2022. The radiotherapy targeted the left supraclavicular region and cervical lymph nodes, with a total dose of 60.2 Gy delivered in 28 fractions. After radiotherapy, the patient was followed up until May 2024, with no evidence of disease progression. The follow-up evaluation included enhanced chest and abdominal CT scans (performed every 2–3 months) and routine laboratory tests, which included complete blood count, biochemistry panels, urinalysis, coagulation tests, and tumor markers such as AFP, CEA, CA-125, HE4, CA199, CA724, cytokeratin 19 fragments, β-HCG, NSE, PSA, and free PSA. The complete treatment timeline is shown in **Figure 4**.

## Discussion

Ovarian-type tumors of the testis are extremely rare, and the origin of these tumors is still debated. However, the majority of authors believe that they arise from Müllerian metaplasia of the peritoneal lining of the tunica vaginalis, the appendix testis, or Müllerian remnants between the testis and the spermatic cord (5, 7–9). This theory is supported by immunohistochemical analysis, which shows positive epithelial markers of Müllerian differentiation, such as PAX-8, WT-1, CK7, EMA, ER, and PR, and negative markers, such as D2-40, PLAP, SALL4, OCT4, Inhibin-a, Calretinin, CK20, and P63 (7, 10). The tumor in our patient exhibited typical pathological characteristics resembling ovarian serous carcinoma, supporting the diagnosis.

Serum CA-125 has been considered a useful diagnostic and prognostic marker, as it is found in ovarian serous carcinomas (1). When our patient developed widespread metastasis in lymph nodes, his serum CA-125 level was found to be elevated. Following the regression of his disease after treatment, his serum CA-125 level decreased accordingly, suggesting that serum CA-125 may be a reliable tumor marker for this disease.

Due to the rarity of these tumors, clinical experience with their management is limited. No consensus or guidelines for treatment exist; however, the majority of scholars recommend using the same treatment protocols for ovarian serous carcinomas in women (1). Radical orchiectomy is typically recommended for primary diagnosis and treatment (11, 12), followed by chemotherapy according to the staging. Although there is no agreement on the best chemotherapy regimen, the combination of paclitaxel and carboplatin is the most commonly reported first-line treatment for testicular serous carcinomas (1, 5, 7). However, treatment outcomes have been variable. Chemotherapy regimens with paclitaxel and carboplatin (1, 7), DCF (docetaxel, cisplatin, and 5-fluorouracil) (13), and estrogen therapy (14) have all demonstrated disease control in some cases. However, some authors have reported that malignant epithelial tumors of the testis are highly chemoresistant (11, 12, 15).

Recently, advances have been made in drug therapy for ovarian carcinomas in women, such as bevacizumab, PARPi, and ADC. These new agents have been infrequently applied to their testicular counterparts. In our patient, chemotherapy with bevacizumab was adopted, resulting in a partial response. This allowed the patient to undergo local treatments, including surgery and radiotherapy, and achieve long-term survival. To the best of our knowledge, this is the first reported case of using a combination of bevacizumab for this rare disease, suggesting that bevacizumab may be a promising option for the treatment of ovarian-type testicular tumors.

## Conclusion

Ovarian-type tumors of the testis, although extremely rare, can be treated using protocols similar to those for ovarian serous carcinomas. In this case, a combination of chemotherapy and bevacizumab resulted in a favorable outcome, offering new insights into treatment strategies for this rare malignancy. Given the challenges in diagnosis and the lack of standardized treatment regimens, further studies are necessary to explore the efficacy of bevacizumab and other advanced therapies in the management of ovarian-type testicular tumors.

## Data Availability

The raw data supporting the conclusions of this article will be made available by the authors, without undue reservation.
